# Bioengineering Human Pluripotent Stem Cell-Derived Retinal Organoids and Optic Vesicle-Containing Brain Organoids for Ocular Diseases

**DOI:** 10.3390/cells11213429

**Published:** 2022-10-30

**Authors:** Peggy Arthur, Laureana Muok, Aakash Nathani, Eric Z. Zeng, Li Sun, Yan Li, Mandip Singh

**Affiliations:** 1 College of Pharmacy and Pharmaceutical Sciences, Florida A&M University, Tallahassee, FL 32307, USA; 2 Department of Chemical and Biomedical Engineering, FAMU-FSU College of Engineering, Florida State University, Tallahassee, FL 32306, USA; 3 Department of Biomedical Sciences, College of Medicine, Florida State University, Tallahassee, FL 32306, USA

**Keywords:** human induced pluripotent stem cells, retinal organoids, assembled organoids, ocular diseases, extracellular vesicles

## Abstract

Retinal organoids are three-dimensional (3D) structures derived from human pluripotent stem cells (hPSCs) that mimic the retina’s spatial and temporal differentiation, making them useful as in vitro retinal development models. Retinal organoids can be assembled with brain organoids, the 3D self-assembled aggregates derived from hPSCs containing different cell types and cytoarchitectures that resemble the human embryonic brain. Recent studies have shown the development of optic cups in brain organoids. The cellular components of a developing optic vesicle-containing organoids include primitive corneal epithelial and lens-like cells, retinal pigment epithelia, retinal progenitor cells, axon-like projections, and electrically active neuronal networks. The importance of retinal organoids in ocular diseases such as age-related macular degeneration, Stargardt disease, retinitis pigmentosa, and diabetic retinopathy are described in this review. This review highlights current developments in retinal organoid techniques, and their applications in ocular conditions such as disease modeling, gene therapy, drug screening and development. In addition, recent advancements in utilizing extracellular vesicles secreted by retinal organoids for ocular disease treatments are summarized.

## 1. Introduction

Organoids, also known as “mini-organs”, grow in a three-dimensional (3D) environment in vitro with mini-clusters of cells that self-organize and develop into functional cell types mimicking the structure and function of an organ in vivo. Organoids can be obtained in vitro from embryonic stem cells (ESCs), induced pluripotent stem cells (iPSCs), neonatal or adult stem cells [1,2] via a mechanism comparable to the development process of an organ with unique architecture. Self-organization occurs within the organoids by spatially constrained lineage commitment and cell sorting, which necessitates the activation of multiple signaling pathways (e.g., Wnt) mediated by intrinsic cellular components or extrinsic environments including the extracellular matrix (ECM) and media [3]. Organoids have several advantages over traditional approaches, including the ability to mimic the near-physiological organ system by partially restoring the structural and functional characteristics of the actual organ. The organoids bear significant cellular heterogeneity, architecture barriers similar to in vivo tissues, and intercellular communication machinery, providing an analogous developmental model to extend direct access for targeted studies [4,5,6].

Various therapeutic advances in ophthalmology have been explored for ocular diseases recently, including photoreceptor cell replacement and retinal sheet transplantations [7,8]. Stem cell therapy has the potential to solve the issues of restricted availability and ethical difficulties related to the usage of fetal retinal tissues, due to the generation of human ESCs and iPSCs and the ability of the researchers to generate retinal cells, tissues, and organoids from these pluripotent stem cells [9,10,11]. Retinal organoids (ROs), in a way similar to brain organoids [12], mimic various aspects of retinal development, which can advance our knowledge of normal retinogenesis and allow for ocular disease modeling in vitro. Human iPSCs have been shown to generate optic cups, optic vesicles, photoreceptors [11,13,14] and retinal pigment epithelium (RPE) cells [15,16,17]. To generate ROs with neuroepithelial cells, iPSCs proliferate and aggregate into neurospheres, which include forebrain progenitor cells and optic vesicles. Retinal ganglion cells, amacrine cells, and horizontal cells then emerge from the latter, followed by photoreceptors (containing rods and cones), bipolar cells, and Muller glial cells [14]. Ongoing preclinical and clinical studies based on these stem cell-derived retinal cells draw our attention to use ROs in treating ocular diseases. Gene-editing techniques, such as CRISPR/Cas9 technology, enable the creation of isogenic controls from patient-derived iPSCs and the introduction of disease-causing mutations in healthy cells, resulting in the creation of novel disease models [18,19,20]. In addition, some 3D human brain organoids containing neural–retinal sections produced from human iPSCs offer an unrivalled opportunity to investigate retinal diseases as well as the complexities of brain development and disorders in recent years [21,22,23].

One of the biggest obstacles to fully understanding and treating ocular diseases is the lack of available preclinical models. Mouse models have been able to provide incredibly vital information. However, it is possible that some of the obtained information might not be able to be translated to humans. The need for more reliable models has led to a recent uptick in studies involving ROs [12]. Human-iPSCs-derived ROs have many advantages such as easy accessibility and high stability. Additionally, transcriptome profiling of ROs has shown that the cell types, specific cell differentiation markers, retinal disease genes, and alternative mRNA splicing of maturing ROs are similar to those of human retinas [24]. RO modeling for ocular disease therapeutics has been designed in the past decade. Although ROs do not recapitulate the exact in vivo vasculature and nervous system conditions, they have been spotlighted for advanced therapeutic models lately. Using next-generation sequencing methods, researchers compared growing ROs to the human retina [25,26,27,28].

**Figure 1:**
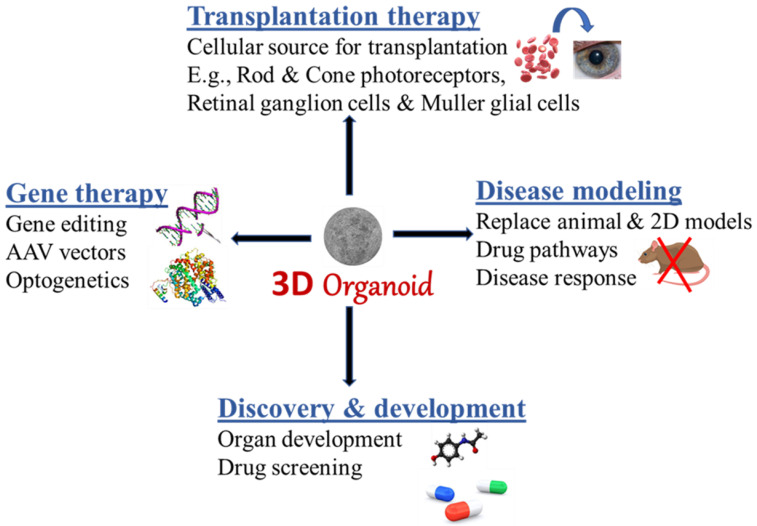
Schematic summary of applications of brain and retinal organoids derived from human pluripotent stem cells.

Various drugs and therapeutics have been developed to treat ocular diseases, most of which, however, only slow the progression or minimize the ocular tissue degeneration. Unfortunately, this means that many patients cannot regain full vision after the treatments. This is because the retina is a highly complex vascularized tissue that contains at least 60 functionally different cell types. All these cell types have to successfully work together in order for visual information to travel from the eye into the brain. There is, however, hope for a solution with the growing interest in stem cells. This review provides recent advances in brain and retinal organoid techniques and their applications in ocular diseases. The use of extracellular vesicle (EV)-based treatments produced from retinal organoids are also highlighted. In summary, ROs are useful for disease modeling, high-throughput drug screening, and possibly a great replacement or alternative to animal models for ocular disease-related applications as highlighted in Figure 1.

## 2. Bioengineering Retinal Organoids from iPSCs

With suitable apical and basal polarity and time-dependent self-patterning of key cell types, ROs closely resemble in vivo retinogenesis and retinal morphology [11,28,29]. ROs can contain most retinal and neuronal cell types, including rods and cones, ganglion, bipolar, horizontal, amacrine, and Müller cells, mimicking the retina’s lamination with most photoreceptor nuclei in the outer nuclear layer (ONL) and a few misplaced cones in the inner nuclear layer [25,26,27].

### 2.1. Differentiation of iPSCs into Retinal Organoids

The patterning of the early eye field is governed by finely tuned regulatory networks of signaling pathways and transcription factors [30]. Retinogenesis commences with the specification of the forebrain neuroectoderm. The rostral neuroectoderm undergoes distinct morphological changes, including lateral expansion of bilateral eye fields to generate the optic vesicles (OVs), which invaginate and become the optic cups [31]. Landmark research using fetal and neonatal tissues has revealed unique insights into human retinal development not found in model organisms [32,33,34]. iPSCs can be directed to self-organize into 3D OV or optic cup structures by supplying suitable systemic and exogenous stimuli [11,35]. Several research groups have demonstrated the ability to generate different retinal cells utilizing hPSCs [13,36,37].

**Figure 2:**
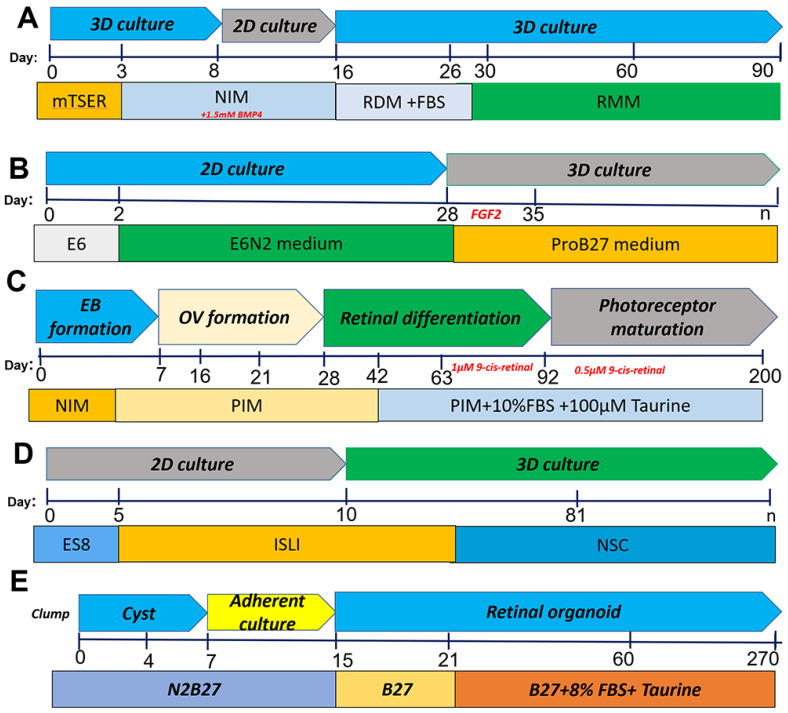
Summary of various protocols (**A**–**E**) for retinal organoid differentiation from human induced pluripotent stem cells. (**A**). The protocol of using 3D–2D–3D culture in retinal differentiation medium. Chichagova et al., 2017 [43]. (**B**). The protocol of using 2D–3D culture in ProB27 medium. Reichman et al., 2019 [44], (**C**) Long-term retinal differentiation with embryoid bodies, which shows the photoreceptor maturation. Kaya et al., 2019 [45]. (**D**) The protocol of using 2D–3D culture in NSC medium. Zhu et al., 2018 [46]. (**E**) Long-term retinal differentiation starting with cyst and adherent culture for organoid maturation. Kim et al., 2019 [47]. Neural induction medium (NIM), retinal differentiation medium (RDM), retinal maintenance medium (RMM), fetal bovine serum (FBS), photoreceptor induction medium (PIM), fibroblast growth factor (FGF), neural stem cell (NSC).

Extrinsic chemical agents are often used to control specific signaling pathways and recapitulate the in vivo microenvironment where retinal cell specification occurs when iPSCs are differentiated into retinal cells in vitro. Combinations of bone morphogenetic protein (BMP), Wnt, Nodal, and Notch pathway inhibitors (such as Noggin, DKK1, Lefty A, and DAPT respectively), numerous growth factors, including insulin-like growth factor (IGF)1, basic fibroblast growth factor (bFGF), activin, sonic Hedgehog (SHH), and triiodothyronine (T3) [38,39,40,41,42] are used in the differentiation protocols to induce retinal progenitor lineage specification (Figure 2 and Table 1). Exposure to native retinal cells in co-culture systems or several exogenous factors such as Noggin, DKK1, DAPT, IGF1, bFGF, T3, SHH, retinoic acid, and taurine [38,39,40,41,42] has also been required for further differentiation into photoreceptors.

**Table 1:** Summary of differentiation protocols for hPSC-derived retinal organoids.

Cell Source	Differentiation Methods	Characterizations	Reference
IPSC (NCL1 from NxCell, Inc.)	Small molecule protocol: IWR1 (a Wnt signaling inhibitor) SB431542 (a TGFβ signaling inhibitor) LDN193189 (a BMP signaling inhibitor) IGF1; neural stem cell (NSC) medium	Pan-photoreceptor markers *OTX2, CRX*, and *RECOVERIN* in the outer layer of RO; retinal stem cell, ganglion cell and amacrine cell marker, *PAX6*; expressed markers of retinal ganglion cells, *BRN3* After 12 weeks of differentiation and s expressed several pan photoreceptor markers, including *BLIMP1, RECOVERIN*, and *AIPL1*	[46]
hiPSC lines PEN8E, 901, 902	Scrapping method for RO generation; formation of EB from hPSC, neural induction medium (NIM),Scraping of adherent cells to form free floating OV, retinal induction medium (B27), 1% NEAA, 1% GlutaMAX	New scraping method displayed morphology similar to that of the dissected organoids and recapitulated the temporal development of the in vivo retina. Retinal ganglion cells (RGCs), horizontal or amacrine cells, *ARL13B* and basal body marker *PCNT*, photoreceptor cilia and basal bodies at day 200.	[48]
Three hiPSC lines, IMR90-437 (WiCell), CB-iPSC6.238 and KA.1	Blebbistatin for aggregate formation induction (NIM), taurine and 2 mM GlutaMAX. Treatment with retinoic acid	Formation of optic cups; expression of a group of transcription factors including *PAX6, RX, LHX2, SIX3*, and *SIX6*, while the surrounding anterior neuroepithelial cells express *PAX6* and *SOX*,	[49]
Human iPSCs	Based on the concept that IGF1 signaling, in conjunction with retinoic acid and triiodothyronine, crucial for retinal development NIM+ BMP4; RDM +FBS; RMM	Photoreceptors, bipolar, horizontal, amacrine, Müller, and retinal ganglion cells develop a thick layer of neuroepithelium in the organoids at 22 weeks	[43]
iPSCs from *Nrl*-GFP mouse	10-day differentiation in static culture followed by maturation in RWV bioreactor (3D); RMM/N2; B-27-VitA, IGF1, NEAA	Transcriptome analysis shows ROs better mimic the spatiotemporal development observed in retinogenesis in vivo; when compared to static cultures, RWV organoids demonstrated rapid differentiation; expression of Müller glia cells and rod photoreceptor markers such as BRN3 and opsin 1 short-wavelength-sensitive	[50]

Three-dimensional organoids have several advantages over 2D or static cultures. Microenvironments, such as biochemical and biophysical signals, as well as cell–cell and cell–matrix interactions, influence the growth and development of 3D organoids derived from stem cells [51,52]. In static suspension organoid cultures, efficient differentiation can be hampered due to diffusion-limited transport of exogenous elements (e.g., nutrients, oxygen) and lack of biophysical stimulation, which may prevent the generation of desirable cell types.

Chichagova et al. used a differentiation technique (Figure 2A) to generate ROs from human iPSCs that contain all main retinal cell types and are light responsive [43]. Human iPSCs were differentiated in 96-well plates, allowing for potential high-throughput production of organoids that can be employed for various purposes, including studying human retinal development, disease modeling, and chemical screening. The differentiation strategy is based on the notion that IGF1 signaling, together with retinoic acid and triiodothyronine, is critical for retinal development. The organoids generate a thick layer of neuroepithelium after 22 weeks in culture, containing photoreceptors, bipolar, horizontal, amacrine, Müller, and retinal ganglion cells (RGCs) [43]. 

Reichman et al. developed a new retinal differentiation method (Figure 2B) without xenogeneic products that allow for the self-formation of neuronal retina-like structures and the creation of RPE cells from confluent human iPSCs [44]. This protocol involves using E6N2 medium (i.e., Essential 6 medium plus 1% Cell Therapy Systems N2 supplement). On day 28, self-formed Ros were scraped off from surrounding cells and grown as floating structures in ProB27 media supplemented with 10 ng/mL of human FGF. Around day 42, the distal section of the neuroepithelium became pigmented, and spherical organoids grew larger. Retinal progenitor cells can be differentiated into the photoreceptor lineages, as evidenced by an increase in RCVRN and CONE ARRESTIN (CAR) expression [44]. RPCs could be differentiated into all four types of retinal cells using floating cultures of isolated structures, and transplantation-compatible CD73+ photoreceptor precursors can be produced in less than 100 days. These xeno-free culture conditions enable the preservation of mature cones and rods in retinal organoids with photoreceptor ultrastructures for up to 280 days. Furthermore, it was feasible to cryopreserve both hiPSC-derived retinal organoids and dissociated retinal cells while maintaining their phenotypic properties and the preservation of CD73+ photoreceptor progenitors [44]. Parallel to the development of neural retina, RPE cells can easily be multiplied, passed through different stages, and frozen while maintaining RPE phenotype. With increased CRX, RECOVERIN (RCVRN), and CAR expression throughout the floating culture, RPCs could be committed into the photoreceptor lineage. After 100 days, the expression of genes specific to mature photoreceptors, such as *RHODOPSIN (RHO)* and *OPSIN (OPS)*, was observed. At day 49, immature photoreceptors immunoreactive for *CRX, OTX2*, and *RCVRN* can be seen, which increased their expression overtime [44].

During development, ROs derived from human iPSCs show many variations. Kaya et al. evaluated the neuronal retina’s developmental maturity in vitro and devised new techniques [53]. Multiple bioinformatics methods were utilized to compare transcriptome analyses of growing ROs differentiated from human ESCs and human iPSCs. The gene expression profiles were used to reveal developmental heterogeneity in organoids, and the combined comparison with human fetal and adult retinal transcriptome data were able to evaluate the molecular stage in the retinal tissue development [45]. As shown in Figure 2C, iPSCs were plated in ultra-low attachment culture plates in E8 media with a ROCK inhibitor Y27632 to generate embryoid bodies (EBs). The medium was replaced with a photoreceptor-induction medium (PIM). When OVs with neuroepithelium morphology appeared during days 21-28, the regions were excised and transferred to suspension culture in PIM. At day 63, the PIM was supplemented with 1 M retinoid, which was gradually decreased to 0.5 M by day 92. The effects of 9-cis retinoid acid (9CRA) on one set of organoids were compared to those of all-trans retinoic acid (ATRA). The addition of 9CRA, rather than the commonly used ATRA, accelerated rod photoreceptor differentiation in organoid cultures, with higher rhodopsin expression and more mature mitochondrial morphology visible by day 120 [45]. 9CRA-supplemented organoids showed faster rod photoreceptor development and identification of rhodopsin protein. While ATRA only binds to retinoic acid receptors (RARs), 9CRA is a potent agonist for both retinoid X receptors (RXRs) and RARs [53,54]. It has been demonstrated that retinoic acid stimulates the growth of photoreceptors [55,56,57] and increases the expression of the rod differentiation factor NRL [58]. Advancements in human ESC [9] and iPSC generation [45] as well as the bioengineering approaches of 3D organoid cultures [11,59] have revolutionized human tissue development studies, facilitated individualized disease modeling, and revitalized the field of regenerative medicine [60,61,62].

**Figure 3:**
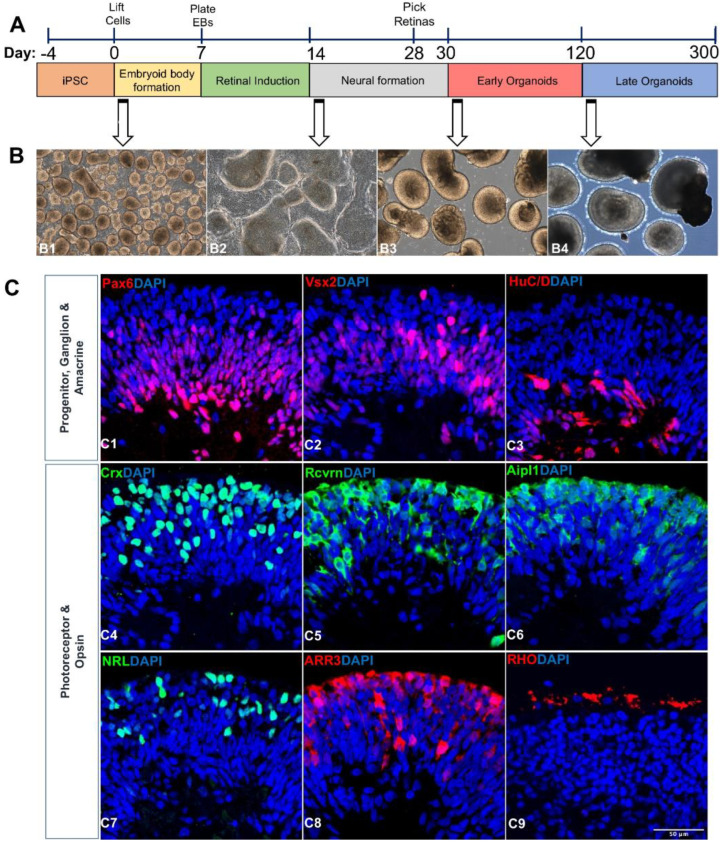
**Retinal organoid differentiation and characterization** [68]. (**A**) Schematic illustration showing the differentiation of retinal organoids from hiPSC and timeline representation of the early (Day 30–120) and late organoids (Day 121–300) for exosome preparation. (**B**) Phase contrast microscopic images showing the morphology of retinal organoids at different time points of differentiation (Day 2, B1; Day 25, B2; Day 45, B3; Day 200, B4). Scale bar, 200 µm. (**C**) Confocal images of 90-day old retinal organoids (C1–C8) stained for retinal progenitor cells *(Pax6*, C1; Vx2, C2), ganglion and amacrine cells (Brn3a, C3), pan photoreceptors *(Crx,* C4; *Rcvrn*, C5; *Aipl1*; C6), rod photoreceptor (NRL; C7), and cone photoreceptor (*ARR3*, C8). Image of 200-day old retinal organoids showing the staining of rhodopsin protein indicating the maturation of rod photoreceptor (*Rho*, C9). Scale bar, 50 µm. Figure reproduced from Arthur et al., (2022) under a Creative Commons license.

By blocking Wnt and BMP signaling, hPSCs can adopt characteristic features associated with all main stages of the early eye and retinal development while adhering to an anticipated timeframe for human retinal development [63,64]. A component of the primitive anterior neuroepithelium forms the eye field in the first few weeks of human development [65,66,67]. These cells express various eye transcription factors, such as *Pax6, Rx*, *Six3, Six6* and *Lhx2*. Despite maintaining an anterior neural identity, the majority of the early Pax6+/Rx+ population did not later adopt cellular phenotypes of the optic vesicle or optic cup [13]. The enriched Pax6+/Rx+ cell population, therefore, most closely mirrored a primitive stage of the development of the human eye field, which occurred before the emergence of committed retinal progenitors [13]. A small molecule-based strategy was used that included IWR1 (a Wnt signaling inhibitor), SB431542 (a transforming growth factor (TGF) signaling inhibitor), LDN193189 (a BMP signaling inhibitor), and IGF1 to stimulate retinal differentiation (Figure 2D) [46]. This work was adapted as a low-cost retinal differentiation alternative by replacing DKK1 (a Wnt signaling inhibitor) and Noggin (a BMP signaling inhibitor) with compounds that target these pathways and have been shown to cause retinal differentiation. A polarized pattern of Cone-rod Homeobox (CRX)+ and OTX2+ cells was found in the outer layer of the ROs, indicating the photoreceptor development and lamination. ROs showed clear signs of maturation after 12 weeks of differentiation and most of the cells in the outer layer of the organoids expressed several pan-photoreceptor markers, including *OTX2, CRX, BLIMP1, RECOVERIN (RCVRN),* and aryl hydrocarbon receptor-interacting protein-like 1 (*AIPL1*) [46]. A N2/B27 protocol was also used to generate retinal organoids (Figure 2E) [47]. A study by Capowski et al. shows the reproducible generation of retinal organoids from 16 hPSC lines, reducing inconsistencies among cell lines. Three stages of retinal organoid growth could be easily distinguished morphologically by optical coherence tomography for >175 days. Stage 1 organoids contained neural retina progenitors (NRPCs) within a growing outer neuroblastic layer, along with a large number of RGCs and uncommon starburst amacrine cells (SACs) that formed an inner RGC layer as well as a discontinuous inner plexiform-like layer [27]. NRPCs experienced increasing differentiation into PRs, horizontal cells, and amacrine cells during Stage 2, a transitional stage during which the discrete RGC layer gradually eroded. Stage 3 was characterized by the development of PR outer segments in addition to other characteristics of the advanced retinal organization and by the ongoing loss and/or disarray of the different inner retinal cell types and layers. Stage 3 is the best for simulating PR-based disorders since retinal organoids have reached an advanced state of PR organization and development, including the production of the inner and outer segments and the outer nuclear and plexiform layers [27]. Our own study also developed retinal organoid differentiation protocols for the investigation of cell–cell communications [68] (Figure 3).

### 2.2. Transcriptome Analysis of Retinal Organoids

Rigorous transcriptome analysis and comparisons with human fetal and adult retina are required to establish ROs as viable models for retinal investigations. Single cell RNA sequencing (scRNASeq) has been used in human retinal tissues and organoids in a few pioneering investigations [69,70,71]. These studies have primarily focused on using reporters or bait genes to enrich specific cell types. The presence of several retinal cell types and their progressive emergence during the differentiation were shown using scRNASeq on ROs. The results demonstrated the identification of different cell types that arise within complex organoids, allowing for deep molecular and temporal systematic analyses as well as for close comparisons between in vitro produced retinal tissues and in vivo organogenesis [72]. Phillips et al. used unbiased algorithms to profile scRNA-seq of long-term ROS and capture photoreceptor gene connections but were unable to uncover individual cell clusters [70]. Cui et al. investigated the time points of retinal development-related biological processes during the formation of the native retina and ROS in mice and humans using bioinformatics-based transcriptome analysis [72]. From these results, Ros develop at a slower rate than native retinal cells, and cell proliferation takes precedence at the early stages as well as neuronal differentiation. In human ROs, the fatty acid metabolic process and mitochondria-related genes are gradually elevated, while the glycogen catabolic process and activin receptors are gradually downregulated. In mouse ROs, however, these developmental patterns are the opposite [72].

### 2.3. Bioengineering Optical Vesicle-Containing Brain Organoids from iPSCs

Three-dimensional brain organoids are innovative tools for studying human neurodevelopmental diseases (NDDs), allowing for noninvasive investigation of patient-derived human tissues [27,73,74]. Parkinson’s disease [75], Alzheimer’s disease [76], amyotrophic lateral sclerosis [77], and other neurological disorders [78] are pathologically and clinically diverse conditions characterized by the accumulation of misfolded proteins and the loss of functional neurons in the affected regions of human brains [79]. 

**Figure 4:**
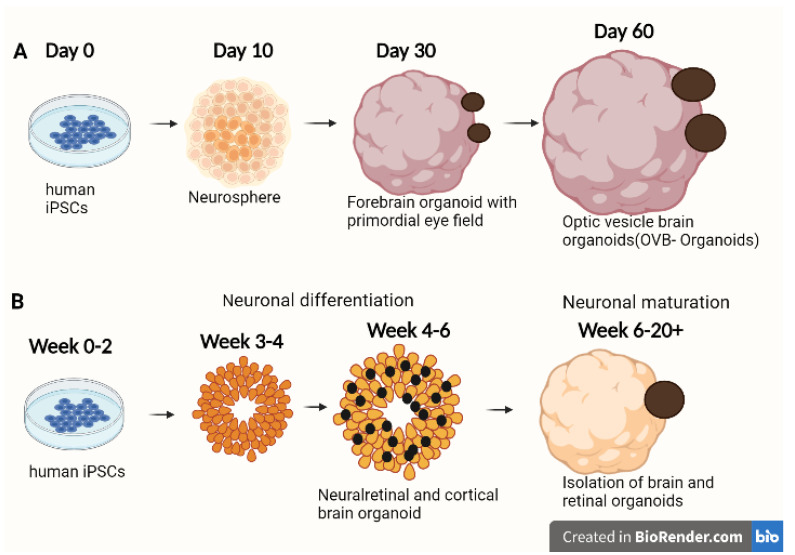
Differentiation protocols of optic vesicle-containing brain organoids for ocular applications. (**A**) Differentiation of iPSCs to brain organoids with optic vesicle regions (OVB), Elke et al., 2021 [83]. (**B**) Differentiation of iPSCs to complex organoid models created by the generation of both retinal and forebrain cortical organoids, Milan et al., 2022 [23]. These organoids can help to study brain–eye interactions during embryo development, model congenital retinal disorders, and generate patient-specific retinal cell types for personalized drug testing and transplantation therapies.

More than 200 years ago, Pander discovered that the retinal anlage grows laterally from the forebrain’s diencephalon, projecting as an OV followed by Huschke, revealing that the distal region of the diencephalon invaginates to build the OV [80,81]. Therefore, cerebral organoids might be useful in ocular diseases besides NDDs. Adelmann supported Pander’s discovery that OVs originate from the diencephalon during embryogenesis through a multistep organogenesis process [82]. In 2021, a study showed that brain organoids have the inherent potential to self-organize fundamental sensory structures associated with the forebrain in a topographically constrained manner, allowing interorgan interactions to be studied within a single organoid [83]. The study was aimed to simplify the complexity by demonstrating the creation of forebrain-associated bilateral OVs, cellular variety, and functionality using human iPSC-derived brain organoids. Around day 30, brain organoids started to put together OVs, which evolve into visible structures in the next 60 days. The cellular components of a developing OV, including primitive corneal epithelial and lens-like cells, retinal pigment epithelia, retinal progenitor cells, axon-like projections, and electrically active neuronal networks, were represented by the optical vesicle-containing brain organoids (OVB-organoids). Synapsin I, CTIP-positive myelinated cortical neurons, and microglia were also present in the derived OVB-organoids (Figure 4A). Different light intensities could activate photosensitive activity in OVB-organoids, and light sensitivities could be reset after brief photobleaching. Henceforth, the ability of brain organoids to pattern optic structures might acknowledge inter-organ interaction studies within a single OVB-organoid [83].

Other studies have shown that brain organoids can exhibit an immature retinal-like structure, and the assembloid approach is one way to generate such hybrid organoids in vitro by fusing different cellular origin components of the brain and OVs [84,85]. Furthermore, in NDDs such as Alzheimer’s and Parkinson’s disease, retinal structure and function have been reported [86,87]. A combinational study using both brain organoids and Ros could better understand these disorders [86]. Fernando et al. described a straightforward and cost-effective methodology for producing the neural retina and forebrain cortical brain areas from confluent stem cell cultures (Figure 4B) [23]. A complex organoid system was created by the generation of both retinal and forebrain cortical organoids. This culture, characterized by the production of cortical organoids along with retinal vesicles and RPE cells, was created by adapting a 2D/3D differentiation approach [14]. Neuronal development and synaptic function proteins were expressed in the cortical organoids based on the proteomics analysis. The 3D retinal–cortical-assembled organoids exhibited retinal nerve-like bundles extending into brain organoids.

## 3. Applications of ROs in Various Ocular Diseases

Breakthroughs in the differentiation of retinal organoids and OVB-organoids from patient-specific iPSCs offer promise tailored human disease models and a resource for assessing treatment methods for various ocular diseases (Table 2) [88]. 

**Table 2:** Pluripotent stem cell-derived retinal organoids for disease modeling and development of therapies.

Eye Diseases	Disease Models	Characterizations of Disease Pathology, or Drug Screening	Reference
Glaucoma	ROs from hPSCs with an E50K mutation in the *OPTN*) gene	Optic nerve damage, high pressure in the eye causing pain and sudden visual disturbance; when derived from three-dimensional retinal organoids, retinal ganglion cells with a glaucoma *OPTN* gene mutation exhibit neurodegenerative phenotypes.	[89,90,91,92]
Leber congenital amaurosis	ROs from hiPSCs derived from LCA4 patient carrying a Cys89Arg mutation in *AIPL1*	LCA is a retinopathy causing visual impairment. Patient ROs showed lower levels of *AIPL1* with no retinal degeneration. ROs confirmed findings in animal models of the disease phenotype	[93,94,95]
Stargardt’ s disease	ROs from iPSCs of four STGD1 patients (homozygous for the p.Gly1961Glu variation, compound heterozygous, and two deleterious *ABCA4* alleles)	Symptoms range from modest vision loss with less obvious fundus abnormalities to a more severe cone or cone-rod dystrophies accounting for 12% of IRD-related blindness	[96,97,98,99]
Retinitis pigmentosa	ROs with electrophysiological properties generated from iPSCs from RP patients with different mutations in the RPGR gene.	Loss of vision, complete blindness is uncommon. Pathogenesis of RPGR using patient-specific organoids. Significant abnormalities in photoreceptors were discovered, including decreased cilia length, photoreceptor cell quantity, and expression of photoreceptor-related genes.	[100,101,102]
	RP11 (*PRPF31*-mutated) patient-derived ROs	In comparison to controls, TEM revealed that patient photoreceptors showed an increase in apoptotic nuclei and the presence of stress vacuoles, indicating progressive degenerative characteristics in ROs	[103]
X-linked retinitis pigmentosa	Temporal maturation of CRISPR gene edited *RP2* knockout Ros as well as ROs derived from patients with the same *R120X* nonsense mutation	Significant loss of vision, complete blindness is common in males. *RP2* knockouts that were isogenic match the phenotype of *RP2* patient-derived organoids. Rod photoreceptor cell death occurs in *RP2* null ROs.	[104,105,106]
X-linked juvenile retinoschisis	hiPSCs from patients to study XLRS in a 3D retinal organoid in vitro differentiation system.	Damaged macula responsible for sharp central vision and visual acuity impairment in children. A retinal organoid model produced from hiPSCs recapitulates important XLRS characteristics. RS1 secretion and retinal development are normalized after CRISPR/Cas9 editing	[107,108,109,110]
Retinoblastoma	ROs generated from CRISPR/Cas9-derived *RB1*-null human embryonic stem cells (hESCs)	Eye cancer developed from immature cells of retina. The loss of RB1 enhanced apoptosis and reduced the number of photoreceptors in *RB1*-null ROs. The absence of *RB1* did not, however, result in the formation of retinoblastoma in ROs	[109,111]

### 3.1. Glaucoma

Glaucoma, an optic neuropathy, results in irreversible vision loss and eventual blindness due to the gradual degradation of RGCs [89]. Glaucoma is the most common cause of irreversible blindness in the world [112]. The loss of RGCs and their axons, which leads to distinctive alterations in the optic disc and accompanying visual field impairment, is the hallmark of glaucomatous neuropathy [90,113]. While the optic nerve head (ONH) and retinal nerve fiber layer (RNFL) can be subjectively assessed in glaucoma, the consecutive introduction of several ocular imaging technologies has altered structural assessment in glaucoma eyes [114,115]. Peripapillary sclera behavior is critical in defining the influence of intraocular pressure (IOP) on the ONH, according to engineering models of ocular tissues that characterize IOP-induced effects [116,117,118]. Features associated with scleral anatomy or physiology, such as axial myopia, corneal hysteresis, and corneal thickness, are all risk factors for human glaucoma [97]. The sclera and the ONH are directly impacted by IOP, which has been shown to have deleterious effects on RGCs and their axons in glaucoma [119,120]. IOP stress is conveyed to RGC axons via the sclera and ONH connective tissues, which are a site of glaucoma damage [121].

Glaucoma, caused by an *E50K* mutation in the optineurin (*OPTN*) gene, destroys the RGCs that act as a link between the eye and the brain. The CRISPR–Cas9 gene-editing technique incorporated the *OPTN (E50K)* mutation into an existing hPSC strain, generating a homogeneous gene control from a patient-derived strain. RGC, distinguished from *OPTN (E50K)*-hPSC, showed multiple neurodegenerative defects, including apoptosis, and increased excitatory activity. These results demonstrate the usefulness of *OPTN (E50K)*-RGC organoids as an in vitro model of neurodegenerative diseases that may develop new therapeutic approaches for glaucoma [92]. Hence, RGC organoids from hPSCs serve as a powerful tool for studying cellular mechanisms of human ocular diseases. 

### 3.2. Leber Congenital Amaurosis (LCA)

LCA is a group of rare early-onset retinal dystrophies with severe clinical symptoms that cause vision loss during childhood [93]. LCA is genetically diverse, with mutations found in at least 25 genes, most of which are essential for photoreceptor development or function [122]. LCA, an ocular condition affecting the retinal tissue and causing visual impairment, accounts for approximately 5% of inherited retinopathies [93]. The majority of LCA patients have an autosomal recessive inheritance. Mouse models have helped clarify disease etiology [94] and preclinical therapy development, with the first adeno-associated virus (AAV)-based gene therapy for recessive LCA caused by RPE65 mutations, which is authorized by the United States Food and Drug Administration [123]. Furthermore, animal models are commonly acknowledged as failing to adequately depict the intricacies of human disease, impeding clinical translation. Different kinds of LCA, particularly dominant forms, are currently untreatable. The CRX protein is required for photoreceptor development in the retina [124]. Through its association with the bZIP transcription factor *NRL*, *CRX* regulates the expression of most rod and cone photoreceptor genes. Different retinopathy phenotypes are caused by mutations in the photoreceptor transcription factor CRX, including early-onset vision impairment in dominant LCA.

Loss of opsin expression was discovered as a standard feature in ROs generated from iPSCs of a second dominant *CRX*-LCA patient carrying the *K88N* mutation, which was mitigated by AAV-mediated *CRX* augmentation. Research has shown that gene therapy can be developed for dominant *CRX*-LCA and other *CRX* retinopathies. The modeling of retinopathies in a patient-specific genetic background has been made possible due to the differentiation of iPSCs into 3D ROs [88]. Three-dimensional ROs derived from iPSCs from an LCA type 4 patient carrying a mutation in the gene recapitulated and supported the disclosures in animal models of the disease phenotype [95].

### 3.3. Stargardt’s Disease (STGD1)

The most prevalent inherited macular dystrophy is STGD1, which is caused by abnormalities in the ATP-binding cassette transporter subfamily A4 (*ABCA4*) gene [96]. Depending on the genotype, the symptoms might range from modest vision loss with less obvious fundus abnormalities to a more severe cone or cone-rod dystrophies [97,98]. Early childhood and early adulthood are the most likely times for symptoms to appear. Bilateral central scotomas, dyschromatopsia, and distinctive macular alterations with yellowish flecked lesions at the RPE level in the macula are common symptoms. STGD1 is one of the most frequently inherited retinal diseases (IRDs) caused by biallelic mutations in *ABCA4*, accounting for 12% of IRD-related blindness [125]. 

Allikmets et al. discovered that there are over 1000 mutations in the *ABCA4* gene linked to STGD1 [96]. Due to the high degree of genetic variation, it is difficult to link certain phenotypic characteristics to specific alleles. Although there is considerable phenotypic variation, there are predictable disease patterns in all STGD1 patients. The pathology starts in the macula, affecting the para-foveal and foveal regions before spreading outside to the peripheral retina. PRs and the RPE degenerate over time, resulting in progressive vision loss [126]. Differentiation of human iPSCs from four STGD1 patients (homozygous for the p.Gly1961Glu variation, compound heterozygous, and two deleterious *ABCA4* alleles) to ROs was performed. These patient-derived ROs mimic complex cellular features of the human retina and express mutant *ABCA4* protein in the distal portions of developing PRs, suggesting that they could be used as a functional model to study the physiological consequences and mechanism of *ABCA4* mutations and other retinal degenerative diseases [99].

### 3.4. Retinitis Pigmentosa (RP)

Another incurable ocular disease that causes blindness is RP due to a RPGR gene mutation, which disrupts the molecular mechanisms required for the formation, conservation, usage, or recovery of rhodopsin, either alone or in combination. The direct result is the gradual and complete loss of rod cells [100,127]. RP is a diverse hereditary disorder characterized by progressive retinal degeneration, affecting one in every 3000–8000 persons globally [127,128]. It begins in the mid-fringe and progresses to the macula lutea and fovea [127]. As a result of the primary degeneration of PR rods and subsequent degeneration of cones, RP is also referred to as rod–cone dystrophy, eventually leading to total blindness. Rods appear to be more affected than cones in PRs. The rod–cone sequences explain why patients develop night blindness early in development and then suffer from visual impairment under periodic situations later in life [128].

A study on RPGR gene correction elucidates that PR loss can be reversed in retinal organoids derived from RP patient-specific iPSCs [101], which recapitulate disease pathology due to the *RPGR* mutation and can be used for drug screening [101]. A couple of studies on RP involving a mutation in pre-mRNA processing factors (*PRPFs*) indicate that *PRPF* animal models do not recapitulate the human RP phenotype [129] or only manifest late-onset RPE defects [130]. Therefore, patient-specific ROs were generated using iPSCs from four RP11 patients having severe blindness due to *PRPF31* mutations. CRISPR–Cas9 correction of the *PRPF31* mutation provided a phenotypic rescue of the molecule and the cells. It offered a proof of concept for the effectiveness of the in-situ gene correction using ROs [103]. Late-onset RP has also been studied using the ROs from patients with *PDE6B* gene mutations, which decipher the underlying mechanisms of RP and evaluate new therapies overcoming interspecies variability due to using animal models [102]. A severe form of RP called X-linked RP (XLRP) caused by *RP2* mutations cannot be studied using animal models, as they do not reproduce the severe phenotype. The ROs generated from *RP2* patient-derived iPSCs and gene-edited isogenic *RP2* knockout iPSCs were used as the disease model [104]. Therefore, ROs can model PR degeneration and screen potential drugs to prevent photoreceptor death.

### 3.5. Usher Syndrome

Usher syndrome is a group of autosomal recessive illnesses marked by congenital neurosensory hearing loss, progressive night vision impairment, and RP-related visual field contraction. Some kinds of Usher syndrome can also cause vestibular dysfunction, leading to a loss of balance. It is the most typical form of inherited deaf-blindness, affecting about 1 in 6000 persons globally [131,132]. Type 1 patients are born severely deaf and develop progressive vision loss due to RP before puberty. Vestibular dysfunction causes developmental motor deficits in the majority of type 1 individuals. With RP discovered in adolescence, type 2 patients have mild to moderate congenital hearing loss. Hearing loss in type 3 patients is gradual and post-lingual, whereas RP onset can take up to a decade [133]. The first symptom of RP is night blindness, which is caused by the degradation of rod photoreceptor cells. The most common cause of RP is mutations in the *USH2A* gene, present in 10–15 percent of recessive RP cases and 30–40 percent of Usher syndrome type 2 cases [134,135]. Gou et al. used iPSCs from patients with the *USH2A* mutation to create ROs. This research could be helpful in the molecular diagnosis and screening of RP and Usher syndrome type 2. This study used patient-specific organoids to recreate the pathophysiology of *USH2A* and showed that mutations in *USH2A* function could lead to cellular and molecular disorders [136]. 

### 3.6. X-Linked Juvenile Retinoschisis (XLRS)

X-linked retinoschisis is one of the most prevalent diseases that leads to juvenile macular degeneration in males [137]. Molecular studies have shown that the disease-causing gene, *XLRS1*, is located on chromosome Xp22. This gene encodes for retinoschisin, a photoreceptor and bipolar cell protein that is involved in cellular adhesion and cell-cell contact [107,138,139]. Perifoveal radial microcysts typically develop in the deep nerve fiber layer in the early stages of the disease. Dendritic, opacified retinal arteries, chorioretinal abnormalities, and alterations associated with the vitreous fluid are additional findings in the affected area. Retinal detachment, neovascular glaucoma, and vitreal hemorrhage are the three main side effects of RS [139,140,141]. Early-life visual acuity loss ranges from modest to severe, with some patients attaining legal blindness by their fifth or sixth decade [141,142]. A research group generated hPSCs from patients to study *XLRS* in a 3D RO in vitro differentiation system to establish an effective disease model [108].

### 3.7. Retinoblastoma

Retinoblastoma (RB) is a rare pediatric cancer that accounts for 6% of childhood cancer cases worldwide and is the most common primary intraocular malignant tumor [143]. RB can be inherited or acquired due to germline and somatic mutations in the tumor suppressor *Rb1* gene, respectively [144]. RB is caused by the biallelic inactivation of the *Rb1* gene, situated at the 13q14 region of chromosome 13 [145,146], which leads to an unlimited capacity for proliferation [147]. *Rb*+/− mice were used in early attempts to simulate retinoblastoma but failed to develop retinal tumors after *Rb1* gene mutations [148,149,150]. In the developing murine retina, conditional inactivation of both copies of *Rb1* could not induce retinoblastoma [151,152,153]. Recently, a 3D self-organizing organoid model was generated from chemotherapy-naïve tumors to serve as a drug screening platform [109]. The organoids retained DNA copy-number changes, gene and protein expression, mimicking retinal tumors. These organoids maintained cone signal circuitry (M/L+ cells) and glial tumor microenvironment (GFAP+ cells). The drug response of the organoids was consistent with that of tumor cells in progressive RB [109]. As another example, human retinoblastoma development was modeled using iPSCs from 15 people with germline RB1 mutations [154]. Using retinal organoids, multiple clones from each iPSC line were examined for the retention of the germline mutation using molecular profiling, including whole-genome sequencing. Then, to evaluate tumor development, the retinal organoids were generated and injected intravitreally of immunocompromised mice. The molecular, cellular, and genetic characteristics of the patient-derived retinal organoids recapitulated human retinoblastomas, which sheds light on the molecular causes of RB as well as the RB1 gene inactivation mechanism [154].

### 3.8. Microphthalmia

Microphthalmia is the presence of an eye with more than two standard deviations shorter in axial length than the age-adjusted population means (21 mm in adults and 14 mm in newborns) [155]. In microphthalmia, the eye is smaller in size and may have a smaller corneal diameter; a microcornea is described as having a horizontal diameter of less than 9 mm in a newborn and less than 10 mm in children older than 2 years [155]. Reports have shown that although microphthalmia affects 3.2–11.2% of blind children, there is no treatment available to improve patients’ eyesight. Current management is focused on maximizing the present vision and improving cosmetic appearance [156,157,158]. Microphthalmia is frequently accompanied by additional ocular abnormalities such as anterior segment dysgenesis, ocular coloboma, cataract, or vitreoretinal dysplasia and exhibits considerable clinical heterogeneity [157]. With dominant, recessive, autosomal, and X-linked inheritance, microphthalmia has a complex etiology, although most mutations linked to non-syndromic microphthalmia emerge spontaneously [157,159].

In animal models, the disruption of Visual System Homeobox 2 (*VSX2*) function results in severe eye and retinal abnormalities, such as microphthalmia, decreased neural retina thickness, and ectopic RPE differentiation [160,161,162]. VSX2 is found in the distal OV and is hypothesized to aid in the integration of OV into the NR in vertebrates by transcriptionally repressing OV and RPE-associated gene microphthalmia-associated transcription factor (*MITF*) [160]. Philips et al. (2014) employed hiPSCs from a patient with microphthalmia with an *R200Q* mutation in the *VSX2* homeodomain region to investigate the function of *VSX2* [163]. Before OV generation, there were no variations between *(R200Q)VSX2* and control hiPSCs. Compared to control hiPSC-OVs, *(R200Q)VSX2* hiPSC-OVs showed a considerable growth deficit and a rise in retinal pigmented epithelium (RPE) development at the expense of NR cell precursors. Additionally, *(R200Q)VSX2* hiPSC-OVs could not generate bipolar cells, a distinguishing characteristic previously seen in *VSX2*-mutant animals. In addition, *(R200Q)VSX2* hiPSC-OVs showed a delayed maturity of photoreceptors, which could be remedied by exogenous expression of wild-type *VSX2* at the beginning of retinal differentiation [163]. Retinal organoids derived from hiPSCs were shown to be essential for understanding the intrinsic elements that regulate retinal development and diseases.

## 4. Platforms of Retinal and OVB Organoid Applications 

### 4.1. A Source for Transplantation and Cell Replacement

For the development of novel cell replacement therapy techniques, stem cells and organoids have been increasingly used as a source [164,165,166,167]. Laminated retinal sheets are another option for treating visual loss in those with advanced retinal degeneration. To overlay the deteriorated retina, 3D retinal tissue can be isolated from the organoids and transplanted intactly into the sub retinal region. The difficulty in preserving correct morphology and polarity while surgically implanting the graft into the recipient eye, as well as the existence of interneurons in the graft, which may limit communication with the remaining host inner retina, are the two important drawbacks of this approach. Retinal sheets transplanted into a rat [168] or non-human primate [167] patients, in contrast to dissociated cells, had a higher success rate.

Many retinal disorders begin with the loss of photoreceptors. When this happens while the neighboring retinal layers are still intact, healthy photoreceptor transplantation could be a viable therapy option. Human stem cell-derived photoreceptors are a unique and limitless supply of human cells for transplantation. In mouse models, it was first expected that transplanted photoreceptors would integrate into the ONL of degenerating retinas and improve eyesight [169,170]. ROs from hPSCs have been used as sources for Müller glial cells and RGCs apart from rod and cone photoreceptors in transplantation [171,172,173]. The use of sheets extracted from human ESC-derived ROs as a therapeutic technique for recovering vision in advanced stages of retinal degeneration was investigated [174]. In a retinal organoid model of XLRP, adeno-associated virus (AAV2/5) was used as a vector to transport the *RP2* gene to photoreceptors in gene therapy [104].

### 4.2. A Platform for Drug Screening

To screen novel compounds for pharmacological and toxicological effects, in vitro models of the human retina are necessary. These models should include all retinal cell types and should be electrical physiologically responsive to light [175]. Most pharmacological investigations are currently conducted in vivo using rat models, but it is inefficient due to structural and functional differences between rodents and human retinas. Anatomical differences, such as the absence of a fovea in most animal models (except simian monkeys) but present in humans, limit the translational potential of most animal models. Other commonly used in vitro cell culture models are limited in their ability to mimic the retina’s composition due to the lack of in vivo-like tissue structure [175,176,177,178]. A study by Eade et al. showed that the increased amounts of lipid species (e.g., deoxysphingolipids) in the age-related retinal degenerative disease macular telangiectasia type 2 (MacTel) lead to photoreceptor degeneration [179]. They discovered that fenofibrate, a medicine frequently used to treat high cholesterol and triglycerides, can also protect retinal cells from deoxySL toxicity by drug screening using ROs. ROs could be an excellent in vitro toxicity test model with a variety of metabolic stresses and potential pharmaceutical interventions for future retinal disease treatment discovery [179].

Saengwimol et al. studied the effects of chemotherapeutic agents with retinoblastoma organoids as a screening platform [109]. Challenges of variability and scalability of retinal organoid cultures have thus far limited their use in high-throughput compound screening. Hilgen et al. created a multiwell plate differentiation strategy that improved ganglion cell growth and sensitivity to physiological stimuli while also enhancing the formation of retinal organoids with retinal-pigmented epithelium [175]. The response of iPSC-derived ROs to Moxifloxacin was investigated. It was discovered that photoreceptors were the predominant impacted cell types, as in the adult mouse retina in vivo. In addition, light-responsive ROs can be generated at the size required for pharmacology and drug screening using properly selected iPSCs [175].

## 5. Importance of Extracellular Vesicles Secreted by Retinal and OVB Organoids 

Emerging evidence suggests that extracellular vesicles (EVs), such as exosomes, microvesicles and exomeres, may participate in the pathogenesis of AMD and other ocular diseases [180,181]. EVs have been defined as a double-edged sword since they can both promote disease progression or support homeostasis maintenance [182,183] depending on their cargo. Exosomes can be obtained directly from healthy retinal or OVB organoid cultures as therapeutics and drug delivery vehicles. 

### 5.1. Characteristics of Extracellular Vesicles (EVs)

EVs include apoptotic bodies, microvesicles, or exosomes based on their intracellular origin and size. Membrane blebbing during apoptosis produces apoptotic bodies, which range in size from 50 to 5000 nm and contain biological components such as DNA, RNA, and histone proteins. The fission of plasma membranes produces microvesicles, also known as microparticles or ectosomes ranging in size of 50–1000 nm. Exosomes are nanosized membrane vesicles that are released by almost all cell types and range in size of 30–200 nm [184,185,186,187]. Exosome biogenesis is characterized by the formation of endocytic vesicles from the plasma membrane, inward budding of the endosomal vesicle membrane, resulting in the multivesicular body (MVB), and the release of exosomes derived from the MVB into the extracellular environment when the MVB fuses with the plasma membrane [21]. Exosomes are produced by all cell types in the human body and can be found in all biological fluids, such as blood, urine, tears, and semen. Function wise, exosomes have been shown to take part in various activities in the body depending on their cell origin. These functions include cell-to-cell communication, immune response, inflammatory response, and neovascularization. Exosomes have also been found to play a role in various diseases such as cancer, cardiovascular diseases, and brain diseases [188,189,190]. More specifically, the applications of exosomes include preventing, detecting, and treating various diseases. While interest in exosomes is receiving significant attention, little work has been conducted on the role that exosomes play in ocular diseases [191,192,193,194,195] or their roles in retinal development (Table 3) [196]. The exosomes secreted by healthy organoids have therapeutic benefits and can be used for drug delivery to correct the messages in ocular disease pathology. The exosomes secreted by diseased organoids need to be corrected for the cargo profiles (i.e., proteins and microRNAs) to prevent disease propagation. 

### 5.2. EVs as Drug Delivery Vehicles for Treating Ocular Diseases

**Figure 5:**
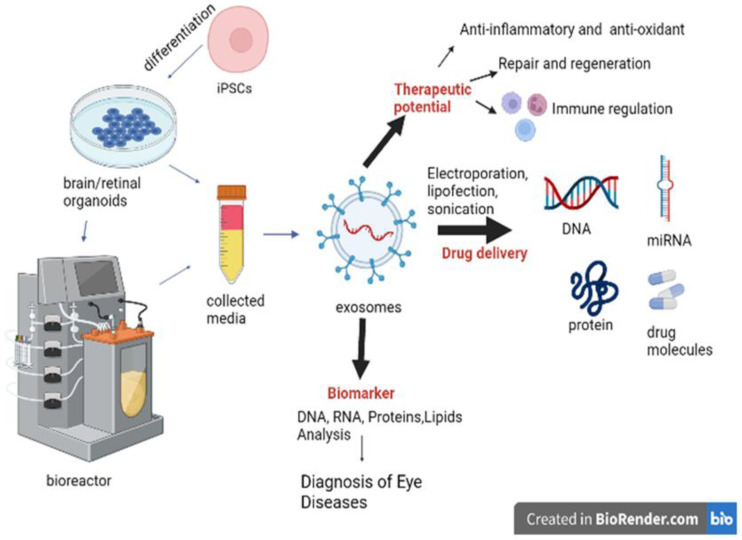
Schematic illustration of various applications of brain and retinal organoids and their derived exosomes in ocular diseases.

Liposomes and polymeric nanoparticles are commonly employed to entrap or encapsulate drug molecules, and these delivery methods are frequently utilized to transport a variety of medicinal molecules, including anticancer, antifungal, and analgesic compounds. However, biocompatibility, stability, long-term safety, and the ability to elude the host immune system while circulating remain key problems [197,198,199,200]. Exosomes have been developed as unique nanoscale delivery vehicles during recent years. Exosomes are ideal nanocarriers as drug delivery vehicles because of several advantages: they do not trigger acute immunological reactions, they have minimal toxicity, they carry the requisite fusogenic qualities, they contain low-uptake machinery, they have high selectivity to the target cells, and they are small and able to cross the blood–brain barrier. Furthermore, tumor tissues tend to store more exosomes than normal tissues [201,202]. By attaching exosomes with tumor-targeting ligands such as proteins, peptides, or antibodies, the selectivity of the exosome delivery platform can be further increased [201,202]. While these abilities make exosomes a promising treatment candidate (Figure 5), there are still many unanswered questions that have yet to be investigated [43,194].

Due to their various functions, it is believed that exosomes could potentially be used for treating ocular diseases. To date, various studies have been conducted on the use of exosomes to treat ocular diseases, involving exosomes derived from mesenchymal stem cells (MSCs), ESCs, and neural progenitor cells [195,203]. A new study published recently by Zhou et al. was the first to show that ROs produced from human iPSCs release exosomes and microvesicles with genetic cargo. The functional RO-derived EVs harboring molecular cargo were released continuously and were related to post-translational modification and human retinal development regulation [196]. It was also shown that the EVs contained small noncoding RNAs cargo including miRNA, tRNA, and piRNA. Further investigation revealed that EVs can transfer their miRNA cargo to human retinal progenitor cells (hRPCs), and the predicted targets were involved in various stages of retinal development. This study revealed that the internalization of RO-EVs by hRPCs and active transport throughout cytoplasmic compartments resulted in the regulation of genes involved in retinal homeostasis and developmental processes, such as nuclear transport, transcription, GTPase regulation, and ganglion and photoreceptor cell differentiation [196]. 

While it has been shown that exosomes contribute to the development of the retina, it has also been observed that exosomes can contribute to the development of retinal diseases, including AMD, diabetic retinopathy, and many others [194]. As mentioned previously, AMD occurs because of an accumulation of drusen in the RPE. Studies have shown that exosomes secreted by donor cells transmit signals that lead to weakening of the RPE’s barrier function, which causes RPE cells to release exosomes that increase autophagy. It is believed that autophagy as well as exosomes from stressed RPE cells play a role in the formation of drusen [43,191,204]. Additionally, it has been observed that these exosomes have surface proteins that are commonly found in drusen. In the case of diabetic retinopathy, it has been observed that exosomes from platelet-rich plasma contribute to retinal endothelial injury by upregulating the TRL4 signaling pathway [191,205]. A recent study by Arthur et al. compares retinal organoid EVs to human umbilical cord (hUC) MSCs in terms of morphological, nanomechanical, molecular, and proteomic characterization. Protein profiling of retinal organoid EVs revealed higher expression of retinal function-related proteins and EV biogenesis marker proteins than hUC-MSC EVs, suggesting that retinal organoid EVs may have a better therapeutic effect on retinal diseases than hUC-MSCs [68].

There are very limited studies about the investigations of EVs from brain organoids besides our previous studies [206,207,208]. The EVs derived from human iPSCs are affected by the differentiation status, lineage specification, and the genetic background of patient-specific iPSCs. EV biogenesis ability may change alongside brain-like tissue development, and the protein and miRNA cargo should be affected by brain regional specificity and the developmental stage. Identification of the specific therapeutic-relevant protein and miRNA cargo in brain-organoid-secreted EVs is critical for treating ocular diseases. The recent discovery of OVB organoids [83] as well as the complex brain and retinal multi-organoids [23] opens the possibility of the use of their EVs for retinal disease therapy. While these studies were able to support the use of exosomes for the treatment of retinal diseases, very few are focused on using exosomes as drug delivery vesicles.

**Table 3:** Summary of retinal organoid-derived extracellular vesicles for ocular applications.

Source of EVs	Method of Isolation	Applications	Reference
Induced-primary RPE (ipRPE) from human retinal organoids	Iodixanol density gradient separation	EVs isolated from RPE derived from ROs contain proteins involved in AMD pathophysiology such as immune response, inflammation, oxidative stress, and drusen composition. This study shows that drusen-associated proteins are present in the RPE-derived EVs and play an active role in drusen growth which is relevant in AMD progression and other retinal diseases.	[204]
hiPSC-derived 3D retinal organoids	Differential centrifugation	The functional 3D RO-derived EVs contains contained small noncoding RNAs cargo including miRNA, tRNA, and piRNA which are released continuously and are related to post-translational modification and human retinal development regulation. The internalization of 3D RO EVs by hRPCs is necessary for the regulation of genes involved in retinal homeostasis and developmental processes, such as nuclear transport, transcription, GTPase regulation, and ganglion and photoreceptor cell differentiation.	[196]
hiPSC-derived 3D retinal organoids	Modified differential ultracentrifugation method with PEG precipitation	Bioreactors were used to upscale secretion of EVs by late time point ROs (>120 days). The EV protein profiling of conditioned medium across the complete development and maturation of retinogenesis can serve as a valuable biomarker for assessing the patient-specific retinal organoids with inherited retinal degenerations. Gene expression analysis by qPCR showed a high expression of exosome biogenesis genes in late retinal organoids derived EVs (>120 day).	[68]

## 6. Limitations of Current Retinal and OVB Organoids

Retinal and OVB organoids have faced considerable challenges due to high variability in organoid formation by different methods [209,210], as well as a lack of blood arteries and an immunological environment. Controlling the organoid formation methods, the use of novel bioreactor system and single cell-RNA sequencing to reduce the variability and understand the cell diversity is critical for the robust generation of the organoids. Although vascularized cerebral organoids have recently been created, establishing an immune niche in organoids remains difficult [211,212] by incorporating brain-resident microglia [213]. Microglial cells play a crucial role in the retina’s natural development because they control neuronal survival and synaptic pruning [214]. Different from retinal cells in organoids, the hematopoietic lineage, which colonizes brain tissue throughout embryonic development, gives rise to microglial cells [215]. 

In brain organoids, astrocytes and neurons spontaneously self-assemble to produce cortical layers mimicking the structure of the human brain [216]. Microglia do not naturally populate brain organoids because they originate from mesodermal lineage [217,218]. Microglia play a function in shaping the CNS at several phases of development, from neurogenesis to the maturation of synapses and neuronal networks, in addition to the roles in immunity [215,219,220]. A thorough single-neuron analysis of the electrophysiological processes that develop in brain organoids after including microglia-like cells was performed [221]. Neurons in organoids enriched with microglia responded to a depolarizing stimulation with persistent, repeated firing. In contrast, neurons in organoids without microglia were more prone to single spiking and adaptation. Neurons co-matured with microglia-like cells displayed a greater diversity in the expression of different neuronal currents, including a prominent post-inhibitory low-threshold current that led to post-inhibitory spiking [221]. This action was attributed to T-type calcium channels, which have been associated with many functions, including synaptic plasticity and neuronal differentiation [221]. Cerebral organoids may serve as a human in vitro platform for research on microglia-neuron interactions since microglia-like cells within the organoids enhance neuronal maturation and recreate some elements of microglia-neuron co-development in vivo.

In addition, RPE development and functioning around PRs is lacking in RO cultures. RPE plays an important role in the protection and survival of PRs [222], which iss supported by the fact that co-culturing the ROs with RPE increased PR differentiation [223]. In addition, the formation of macula in ROs has not yet been accomplished despite the recent advances in the differentiation of iPSCs to ROs [25]. Although there has been an improvement in the cone-to-rod ratio of photoreceptors [27] compared to the mature retina, the response to light stimulation is relatively low. Mostly, studies of retinal organoids have concentrated on photoreceptor development and diseases that severely affect these cells [49,224,225]. In contrast, studies of RGCs within retinal organoids have drawn far less attention. This is because these cells are less organized within organoids and eventually lose their viability in long-term cultures, which limit the applicability of retinal organoids for disorders that affect RGCs. RGCs are the only connection between the eye and the brain since they are the projection neurons of the visual system [226]. The neurotrophic hypothesis suggests that RGCs depend on post-synaptic targets for vital survival signals, which is one reason for the loss of RGCs within retinal organoids. A study by Fligor et al. uses three-dimensional assembloids as a powerful method to explore the increased survival of stem cell-derived RGCs and the mechanisms behind the long-distance projection of RGC axons into physiologically relevant organs to overcome these inadequacies [227].

One limitation of retinal organoid engineering is the variability in organoid formation due to different formation methods. The differentiation procedure for retinal organoids can have a considerable impact on the cellular composition at different developmental stages [228]. The quantity and quality of mature retinal organoids produced by three different procedures hiPSCs were compared [228]. Standardized retinal organoid development methods are required, with an emphasis on the differentiation technique and the extrinsic differentiation cues, to enable the comparison of experimental applications of retinal organoids across research. In addition, heterogeneity inside and between organoids is prevalent, and variations exist [175]. The epigenetic memory from the parental somatic cells may either promote or inhibit the differentiation of hiPSCs toward a particular lineage [229], and methods that are effective in one hPSC line may not function as effectively in others or across several hiPSC lines [13,175]. To enhance and standardize techniques for the routine differentiation of retinal organoids across many cell lines, a clear need for a deeper knowledge of the mechanisms governing retinal organoid development exists. A study was sought to determine whether the strategies utilized to create EBs at the beginning of differentiation affect the ability of the ensuing organoid cultures to differentiate into retina [230]. Transcriptional study showed that retinal organoids grouped more strongly by cell type than by derivation method in the fifth week of differentiation, when the events connected with the early phases of retinal histogenesis are taking place. The effects of cell line background and EB generation and maintenance on the development of retinal organoids from hPSCs were investigated [230]. The findings showed that cell line-specific characteristics predominated among the factors influencing differentiation efficiency in the early phases. In contrast, retinal organoids’ subsequent differentiation and development were determined by the EB production method and maintenance conditions. For example, EB generated mechanically under static conditions and medium supplemented with Y27632 during the first 48 hours of differentiation produced the most consistent laminated retinal neuroepithelium containing mature and electrophysiologically responsive photoreceptors [230].

## 7. Conclusions

There are increasing interests in human ROs with the hope of producing functional ROs that completely recapitulate the human retina. Significant progress has been made for the differentiation of ROs from human iPSCs. The discovery of OVB organoids has promoted the interests for applications in ocular diseases. ROs and OVB organoids are helpful tools for disease modeling, high-throughput drug screening, and an alternative to animal models. The in vitro models for disease studies and drug screening are more cost-effective as alternatives to animal models. Based on current reports on the therapeutic benefits of exosomes in ocular diseases, exosomes derived from ROs and brain organoids have great potential for drug delivery and treating ocular diseases. However, there are still challenges that are yet to be overcome, including lack of vasculature and microglia in the organoids, which are essential for maintaining long-term viability and mimicking the native retina. Nonetheless, emerging bioengineering technologies such as oxygen delivery mechanisms, retina-on-a-chip, and 3D bioprinting are under development to resolve these challenges.

## Data Availability

Available upon request.

## Abbreviations

Adeno-associated virus	AAV2/5
ATP-binding cassette transporter subfamily A4	ABCA4
All-trans retinoic acid	ATRA
Aryl hydrocarbon receptor-interacting protein-like 1	AIPL1
Basic fibroblast growth factor	bFGF
Bone morphogenetic protein	BMP
9-cis retinoic acid	9CRA
CONE ARRESTIN	CAR
Cone-rod Homeobox	CRX
Current good manufacturing practices	cGMP
Embryoid body	EB
Embryonic stem cells	ESCs
Extracellular matrix	ECM
Extracellular vesicle	EV
Human neurodevelopmental diseases	NDDs
Human pluripotent stem cells	hPSCs
Human retinal progenitor cells	hRPCs
Human umbilical cord	hUC
Induced pluripotent stem cells	iPSCs
Inherited retinal diseases	IRDs
Insulin-like growth factor	IGF 1
Intraocular pressure	IOP
a Wnt signaling inhibitor	IWR1
Leber congenital amaurosis	LCA
Macular telangiectasia type 2	MacTel
Mesenchymal stem cells	MSCs
Microphthalmia-associated transcription factor	MITF
Multivesicular body	MVB
Neurodevelopmental diseases	NDDs
Neural retina	NR
Neural retina progenitors	NRPCs
Outer nuclear layer	ONL
Optic nerve head	ONH
Optic vesicles	OVs
Optical vesicle-containing brain organoids	OVB-organoids
Optineurin	OPTN
Photoreceptor- induction medium	PIM
pre-mRNA processing factors	PRPFs
RECOVERIN	RCVRN
Retinal ganglion cells	RGC
Retinal nerve fiber layer	RNFL
Retinal organoids	ROs
Retinal pigment epithelium	RPE
Retinitis Pigmentosa	RP
Retinoblastoma	RB
Retinoic acid receptors	RARs
Retinoid X receptors	RXRs
Single cell RNA sequencing	scRNASeq
Sonic Hedgehog	SHH
Starburst amacrine cells	SACs
Stargardt’s disease	STGD1
Three-dimensional	3D
Transforming growth factor	TGF
Triiodothyronine	T3
Visual System Homeobox 2	VSX2
X-linked juvenile retinoschisis	XLRS
X-linked RP	XLRP
